# Metabolic syndrome in youth with bipolar spectrum disorders treated with second-generation antipsychotics: baseline results from the community-based pragmatic MOBILITY Trial

**DOI:** 10.1007/s00787-025-02680-2

**Published:** 2025-04-11

**Authors:** Claudine Higdon, Jeffrey A. Welge, Nancy A. Crimmins, Christina C. Klein, Victor M. Fornari, Michael T. Sorter, Thomas J. Blom, Brian P. Kurtz, Christoph U. Correll, Melissa P. DelBello

**Affiliations:** 1 Northwell, 2000 Marcus Ave, Suite 300, New Hyde Park, NY 11042-1069 USA; 2 Department of Psychiatry at Zucker Hillside Hospital, Glen Oaks, NY USA; 3 Department of Psychiatry, Donald and Barbara Zucker School of Medicine at Hofstra/Northwell, Hempstead, NY USA; 4 Department of Psychiatry, University of Cincinnati College of Medicine, Cincinnati, OH USA; 5 Cincinnati Children’s Hospital Medical Center, Cincinnati, OH USA; 6 Department of Child and Adolescent Psychiatry, Charité, Universitätsmedizin Berlin, Berlin, Germany; 7 German Center for Mental Health (DZPG), Partner Site Berlin, Berlin, Germany; 8 Institute of Behavioral Science, The Feinstein Institutes of Medical Research, Manhasset, NY USA

**Keywords:** Bipolar spectrum disorders, Metabolic syndrome, Second-generation antipsychotics (SGAs), Youth

## Abstract

Metabolic syndrome (MetS) in obese youth in the general population is about 29%. The aim of this study is to identify the baseline prevalence and clinical characteristics of MetS in a sample of overweight/obese youth with bipolar spectrum disorders treated with second-generation antipsychotics (SGAs). The MOBILITY trial enrolled overweight/obese youth 8–19 years of age with a lifetime diagnosis of a bipolar spectrum disorder who were continuing or starting treatment with an SGA (November/2015–December/2022). Baseline height, weight, body mass index (BMI), blood pressure, fasting lipid profile, glucose, hemoglobin A1c, and insulin were collected. Logistic regression was used to estimate the odds of MetS and each of its component criteria and correlations with demographic factors. Of 1565 randomized patients mean age = 13.9 ± 2.8 years, males = 53%, White = 65.4%, Black/African American = 18.5%, multiracial = 9.6%, obese = 65.7%, overweight = 34.3%, MetS status could be determined for 996 patients (63.6%). The overall prevalence of MetS was 32.8% (males = 49.5%, females = 26.0%, p < 0.001), being higher in obese than overweight youth (41.1% vs 4.1%, p < 0.001). The odds of MetS increased rapidly with age in boys (p < 0.001); no association with age was evident among girls. White patients having significantly higher frequency of MetS than Black/African American patients (35.5% vs 24.9%, OR = 1.75, p = 0.003). The prevalence of MetS in this enriched sample was about one-third higher than the MetS prevalence in the general population of obese youth. Over one third of participants had missing metabolic data. These data highlight the need for increased identification and targeted interventions for MetS in youth. *Clinical Trial Registration*: NCT02515773, https://clinicaltrials.gov/study/NCT02515773

**Supplementary Information:**

The online version contains supplementary material available at 10.1007/s00787-025-02680-2.

## Introduction

There are numerous studies showing an increased prevalence of overweight (OW) and obesity (OB) in adults with bipolar spectrum disorder (BSD) [1], although the prevalence of OW and OB in children and adolescents with BSD remains less clear. In the Course and Outcome of Bipolar Youth study published in 2008, 42% of the sample of youth with BSD were OW/OB, which was approximately 15% greater than the national prevalence cited at 34% [2]. Because the prevalence of OW at 16.1% and OB at 19.3% in the general population of youth ages 2–19 years has increased considerably over the course of the past two decades [3, 4], it has been more difficult to discern an increased illness burden in youth with BSD compared to healthy controls. In the National Comorbidity Survey–Adolescent Supplement, adolescents with BSD had similar prevalence of OW at 20.8% and OB at 17.1% to the general population of youth without affective disorders [5] suggesting that the relationship of BSD and OW/OB happens over time [6, 7]. Nevertheless, children and adolescents with BSD are at increased risk for antipsychotic-induced metabolic aberrations including unexpected weight gain and adverse impact on glucose and lipid levels [8–10].

Metabolic syndrome (MetS) is a clustering of cardiometabolic risk factors. OB and central adiposity are hypothesized to be the major underlying risk factors for developing metabolic syndrome (MetS) [11]; other risk factors include diet, age, intrauterine environment, onset of puberty, and genetics, particularly family history of type 2 diabetes mellitus and insulin sensitivity [12]. Central adiposity, the accumulation of fat in the abdominal area, is comprised of subcutaneous fat, which sits under the skin, and visceral fat, which surrounds the organs in the peritoneal cavity [13]. The hormonal action of the visceral fat stores through the excretion of adipokines impairs glucose metabolism, and higher levels of central adiposity, independent of body mass index (BMI), are associated with cardiometabolic diseases including type 2 diabetes, hypertension, heart disease, and dementia [14]. Although there is no consensus definition for MetS in children and adolescents, key elements of MetS are central OB, high blood pressure (BP), dyslipidemia, and hyperglycemia [15]. The International Diabetes Federation and the American Academy of Pediatrics have called for a focus on cardiometabolic risk factor clustering rather than a syndrome per se [15, 16].

The prevalence of MetS increases when youth are OW or OB; in a systematic review of 85 studies in children, the median prevalence of MetS was 3.3% in normal weight, 11.9% in OW, and 29.2% in OB [17]. As with OB, the prevalence of MetS in youth with BSD is higher than in the general population, i.e., 14% versus 6.7% [18].

There are racial and ethnic differences in the prevalence of metabolic syndrome. Specifically, MetS frequencies are greater in Hispanic youth (6–9%) versus White (4–8%) or Black/African American (2–4%) individuals [19]. Black/African American non-Hispanic youth have lower rates of dyslipidemia [20], yet greater insulin resistance and higher BP than White non-Hispanic and Hispanic youth [15, 21]. Compared to Black/African American non-Hispanic and White non-Hispanic youth, Hispanic children have increased dyslipidemia [22–24].

Second-generation antipsychotics (SGAs) are considered first-line treatments for bipolar I disorder in youth [25]. Weight gain is a well-known side effect of SGAs, and the risk for weight gain is even greater in youth than in adults [25, 26]. MetS is a common consequence of SGA treatment in youth [27–29] and can lead to a myriad of health consequences, including insulin resistance, diabetes, atherosclerosis and cardiovascular disease, polycystic ovarian syndrome, nonalcoholic fatty liver disease (NAFLD), musculoskeletal problems, operative and post-operative complications, psychosocial difficulties, and, ultimately, premature mortality [30, 31].

A substantial number of studies have examined MetS in adults with bipolar disorder [28, 29], but less is known about the prevalence of MetS in children, adolescents, and young adults with BSD [18]. This analysis of the Metformin for Overweight and Obese Children and Adolescents with Bipolar Spectrum Disorders Treated with Second Generation Antipsychotics (MOBILITY) (ClinicalTrials.gov Identifier NCT02515773) study aimed to identify the baseline prevalence of MetS in a community-based, pragmatic clinical trial of OW/OB youth with BSD treated with second-generation antipsychotics (SGAs).

The primary outcome for this analysis of the MOBILITY study was fulfilling criteria for MetS, which was defined, using clinically relevant thresholds for body weight and metabolic parameters in pediatric patients [8] and partially adjusted adult criteria from the United States National Cholesterol Education Program’s Adult Treatment Panel III report (NCEP 2001) [32], as ≥ 3 of the following: BMI ≥ 95th %ile for age and sex, either systolic or diastolic BP ≥ 90th %ile for age-sex-height, fasting triglycerides ≥ 110 mg/dL, fasting high density lipoprotein (HDL)-cholesterol < 40 mg/dL, or fasting glucose ≥ 100 mg/dL [8]. There is no consensus definition of waist circumference (WC) measurement sites in children and adolescents [33], and there is little evidence that in routine clinical practice settings WC provides additional benefit over BMI as a metabolic screen for youth [34]. Furthermore, there are measurement errors associated with WC [35]. Given the pragmatic nature of the study design to mimic real-world clinical practice and the difficulties in reliably implementing WC measurements in community-based mental health centers, BMI ≥ 95th %ile for age-sex was used as proxy criterion for WC ≥ 90th %ile for age-sex.

## Methods

A detailed description of the MOBILITY protocol has been previously reported [36]. In short, MOBILITY was a multi-site, randomized, pragmatic trial to compare metformin combined with healthy lifestyle instruction to healthy lifestyle instruction alone over a 2-year period. Below, we describe the protocol elements relevant to the analyses in this report.

### Study participants

Eligible youth (1) were aged 8–19 years inclusive, (2) had a sex- and age-normalized body mass index (BMI) ≥ 85th percentile, (3) had a lifetime clinical diagnosis of a BSD (bipolar I or II, unspecified bipolar or related disorders, disruptive mood dysregulation disorder, cyclothymic disorder, other specified bipolar and related disorders, as well as mood disorder not otherwise specified), and (4) received a new or had an ongoing prescription for an oral regularly-dosed SGA. Patients were excluded if they (1) had been exposed to a total daily dose of 2000 mg of metformin for at least 2 weeks in the past 3 months, (2) had a major neurological or medical illness that may affect weight gain, 3) required a systemic medication that might impact weight or glucose regulation, (4) were pregnant or breast feeding, and/or (5) had a fasting serum glucose ≥ 126 mg/dL or serum creatinine ≥ 1.3 mg/dL on two occasions during screening or in the prior six months, indicating a need for prompt treatment for diabetes or kidney disease.

At baseline, patients had to reside with a caregiver who was able to answer questions (in English or Spanish) about the child. After reviewing study procedures, all participants aged 18–19 years old or legal guardians or representatives from child services of participants aged < 18 years old provided written informed consent, while minors provided written informed assent.

Given the pragmatic trial design, laboratory measures were recommended but not required for study inclusion, as long as the treating clinician judged the patient to be medically stable. Although it was recommended to obtain bloodwork at the time of the baseline visit, laboratory tests collected up to six months prior to baseline were allowed if no other bloodwork was available.

### Study sites

Patients were recruited for this Patient Centered Outcomes Research Institute (PCORI)-funded study and followed at 64 clinical locations (39 community-based mental health centers and 25 sites in or affiliated with academic health centers). Sites were in California, Connecticut, Kentucky, Maryland, Massachusetts, Minnesota, New Jersey, New York, Ohio, Pennsylvania, and Texas.

### Outcomes

The primary outcome for this analysis of the MOBILITY study was fulfilling criteria for MetS as defined above. In cases where only 3 or 4 of these criteria were measured, it was still possible to determine MetS if ≥ 3 observed criteria were positive or ≥ 3 observed criteria were negative. Hemoglobin A1c (HbA1c), low density lipoprotein (LDL)-cholesterol, total cholesterol, insulin, and alanine transaminase (ALT) (a marker of NAFLD) [37] were also collected.

A standard operating procedure for weight and height measurement was made available to each site, and staff were trained in its application. At each site, weight was measured with a Seca scale, model 882, calibrated to the nearest 0.2 kg per manufacturer. Scales were zeroed before each individual measurement, and patients were instructed to remove shoes and any heavy clothing. Weight was recorded to the nearest 0.1 kg. Height was measured with a wall-secured stadiometer. The stadiometer was calibrated to the nearest 0.1 cm according to the manufacturer. Height was measured without shoes while standing on a flat surface with chin parallel to the floor and was recorded to the nearest 0.1 cm. BMI was calculated as (weight(kg)/ height(m)^2^). Normalized BMI (z-score, adjusted for age and sex), to determine OW and OB weight status were calculated using the program provided by the United States Department of Agriculture/Agricultural Research Service Children’s Nutrition Research Center at Baylor College of Medicine (http://www.bcm.edu/cnrc/bodycomp/bmiz2.html) [38–40]. Blood samples were processed and analyzed locally at each site.

### Statistical analysis

Logistic regression analysis was used to estimate the odds of MetS and each of its component criteria with demographic factors. For analyses of HbA1c, LDL-cholesterol, insulin, and ALT, linear models were used. Models included main effects and all interaction terms. To check whether linearity assumptions were reasonable when assessing age effects, the logit-linear model was compared to models having one or more additional polynomial terms. All confidence intervals and p-values were two-sided, with α = 0.05 without adjustment for multiple testing. Adjustments for multiple comparisons were not performed to maximize statistical power in this secondary analysis.

Descriptive statistics on duration of lifetime SGA exposure are reported, but associations of this variable with MetS or its components were not examined because the sample inclusion criteria induced selection bias (Berkson type). All patients became OW/OB due to some combination of SGA exposure, lifestyle factors, and genetics. Those with minimal SGA exposure had become OW/OB due to one or both latter factors, which also influence the other components of MetS. If these unmeasured risk factors were more common among those with minimal SGA exposure, the association between SGA exposure and outcomes would be negatively biased. The group whose MetS status was determinable was not statistically compared to the undeterminable group, as a direct comparison conflates missing data with the criterion values themselves (i.e., identical missing data patterns sometimes but not always allowed MetS to be determined). Instead, the group with ≥ 1 lab-based MetS criteria measured was compared to those without any available laboratory data, since missing laboratory data was the primary reason for undetermined MetS status.

## Results

### Clinical characteristics

**Fig. 1:**
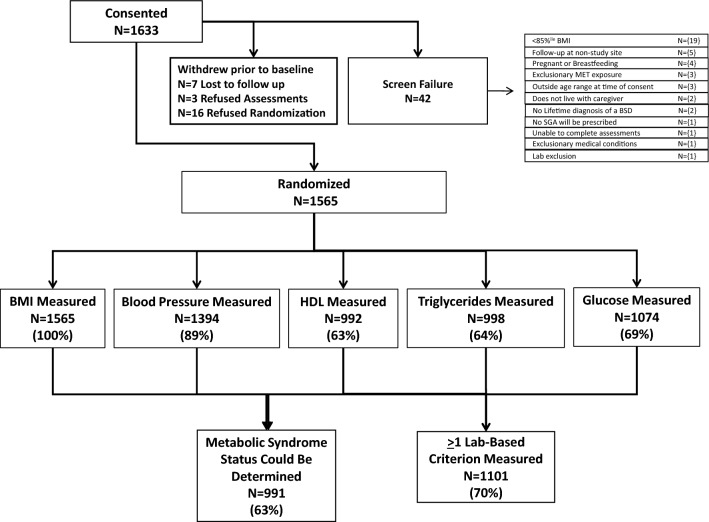
CONSORT diagram of participants consented, randomized, and number with each endpoint

Fifteen hundred sixty-five patients were enrolled in the MOBILITY study between 11/2015 and 02/2022. Mean age was 13.9 ± 2.8 years, with 53% males and 47% females. Table 1 contains demographic information on the total sample and subsample for which MetS status was determinable and descriptive statistics for all endpoints. Figure 1 shows the CONSORT diagram of participants consented, randomized, and number with each endpoint. A majority of the sample, 65.4%, identified as White, 18.5% identified as Black/African American, and 9.6% identified as Multiracial. The proportion of male and female patients (defined as sex assigned at birth) was similar with 53% males and 47% females. The sample was evenly distributed between recruitment from academic medical centers (50.6%) versus community mental health clinics (49.4%). A large majority (91.2%) of the sample was enrolled from outpatient services instead of from inpatient units. At baseline, mean BMI was 29.2 ± 6.1 and BMI z-score was 1.86 ± 0.47.

**Table 1:** Demographic and clinical characteristics of total sample and sample with MetS data

	Overall Sample (N = 1565)				Sample with MetS Data (N = 996)			
Variables	N	%	Mean	SD	N	%	Mean	SD
Age at Randomization (years)			13.9	2.8			13.9	2.8
8–9	189	12.1			121	12.2		
10–11	232	14.8			148	14.9		
12–13	343	21.9			217	21.8		
14–15	381	24.3			257	25.8		
16–17	331	21.2			201	20.2		
18–19	89	5.7			52	5.2		
Sex (assigned at birth)								
Female	736	47.0			461	46.3		
Race								
White/Caucasian	1023	65.4			670	67.3		
Black/African American	290	18.5			180	18.1		
Multiracial	151	9.6			92	9.2		
Does not identify with a race	57	3.6			28	2.8		
Asian	31	2.0			20	2.0		
Native American	6	0.4			3	0.3		
Native Hawaiian/Pacific Islander	2	0.1			2	0.2		
Reported Unknown	5	0.3			1	0.1		
Ethnicity								
Hispanic origin	202	12.9			108	10.8		
Non-Hispanic origin	1361	87.0			887	89.1		
Reported Unknown	2	0.1			1	0.1		
Body mass measures								
BMI (m/kg^2^)			29.2	6.1			29.0	6.0
BMI z-score			1.86	0.47			1.85	0.48
BMI (as % of 95th percentile)			111.2	20.0			110.7	19.4
Obese (≥ 95th percentile for age/sex)	1028	65.7			635	63.8		
Overweight (> 85th or < 95th percentile for age/sex)	537	34.3			361	36.2		

*SD* standard deviation, *BMI* body mass index

A large majority of patients (96.3%) had prior exposure to SGAs, and 48.1% of the sample had more than 1 year of prior SGA exposure (see Table 2a). The most common SGAs ever taken were aripiprazole (59.2%), followed by risperidone (43.2%) and quetiapine (26.5%). Most common SGAs prescribed at baseline were aripiprazole (41.5%), risperidone (24.7%), and quetiapine (17.6%) (see Table 2b).

**Table 2:** (a) Duration of prior Second-Generation Antipsychotic (SGA) exposure, (b) SGAs prescribed at baseline

	N	%
(a)		
None	56	3.6
< 1 Month	81	5.2
1–2 Months	63	4.0
2–3 Months	26	1.7
3–6 Months	109	7.0
6–12 Months	132	8.4
< 1 Year (months unknown)	200	12.8
> 1 Year	753	48.1
Unknown	145	9.3
(b)		
Aripiprazole	649	41.5
Risperidone	386	24.7
Quetiapine	275	17.6
Ziprasidone	68	4.3
Olanzapine	62	4.0
Lurasidone	63	4.0
Paliperidone	54	3.5
Clozapine	13	0.8
Asenapine	9	0.6
Brexpiprazole	3	0.2
Cariprazine	3	0.2
Iloperidone	1	0.1
None	35	2.2
Two or more	52	3.3

### Metabolic syndrome constituents and related laboratory measures

Table 3 summarizes MetS criterion means, standard deviations, and other laboratory measures. The prevalence of each component of MetS was as follows: OB 65.7%, BP 45.1%, triglycerides 41.2%, HDL-cholesterol 30.5%, and glucose 14.3%.

**Table 3:** Metabolic syndrome criterion and other laboratory measures

	N	%
Metabolic syndrome		
Yes	327	32.8
No	669	67.2
Unknown	569	36.4
Numbers meeting each individual criterion		
Obese	1028	65.7
Triglycerides	414	41.2
High Density Lipoprotein (HDL)-Cholesterol	304	30.5
Blood Pressure	629	45.1
Glucose	154	14.3
Individual Criterion	Mean	SD
Triglycerides (mg/dL)	116.4	69.7
HDL-Cholesterol (mg/dL)	46.6	12.0
Systolic Blood Pressure (mmHg)	116.4	13.5
Diastolic Blood Pressure (mmHg)	72.1	10.0
Glucose (mg/dL)	90.2	11.5
Hemoglobin A1c (%), N = 774	5.3	0.7
Insulin (U/mL), N = 759	23.0	27.2
Total Cholesterol (mg/dL), N = 1005	160.8	34.6
Low Density Lipoprotein-Cholesterol (mg/dL), N = 985	91.3	27.2
Alanine Transaminase (U/mL), N = 1016	27.8	30.4

### Metabolic syndrome

The overall prevalence of MetS was 32.8%. Among those with BMI ≥ 95th%ile (already meeting one criterion for MetS), the prevalence was 41.1% (95% Confidence limits 37.6, 44.8), and, for those with BMI between the 85th and < 95th%iles, the prevalence was 4.2% (95% confidence limits 1.3, 7.0).

Table 4 contains associations among the binary criterion variables for MetS. Each criterion was predictive of the remaining criteria (except for the BP criterion, which was not significantly associated with the glucose criterion). Furthermore, meeting each criterion was associated with greater odds of meeting at least two of the other criteria (which would satisfy the criteria for MetS). Elevated triglycerides and low HDL-cholesterol were the most correlated with Spearman correlation = 0.26.

**Table 4:**
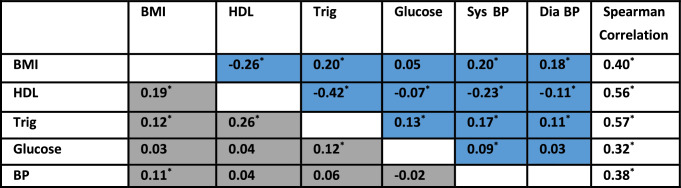
Spearman correlations among metabolic syndrome criteria

*BMI* body mass index, *HDL* high density lipoprotein-cholesterol, *Trig* Triglycerides, *Sys BP* systolic blood pressure, *Dia BP* diastolic blood pressure

^*^p < 0.05

Values in blue = Spearman correlation between measured values

Values in gray = Spearman correlation among binary criterion indicators

**Fig. 2:**
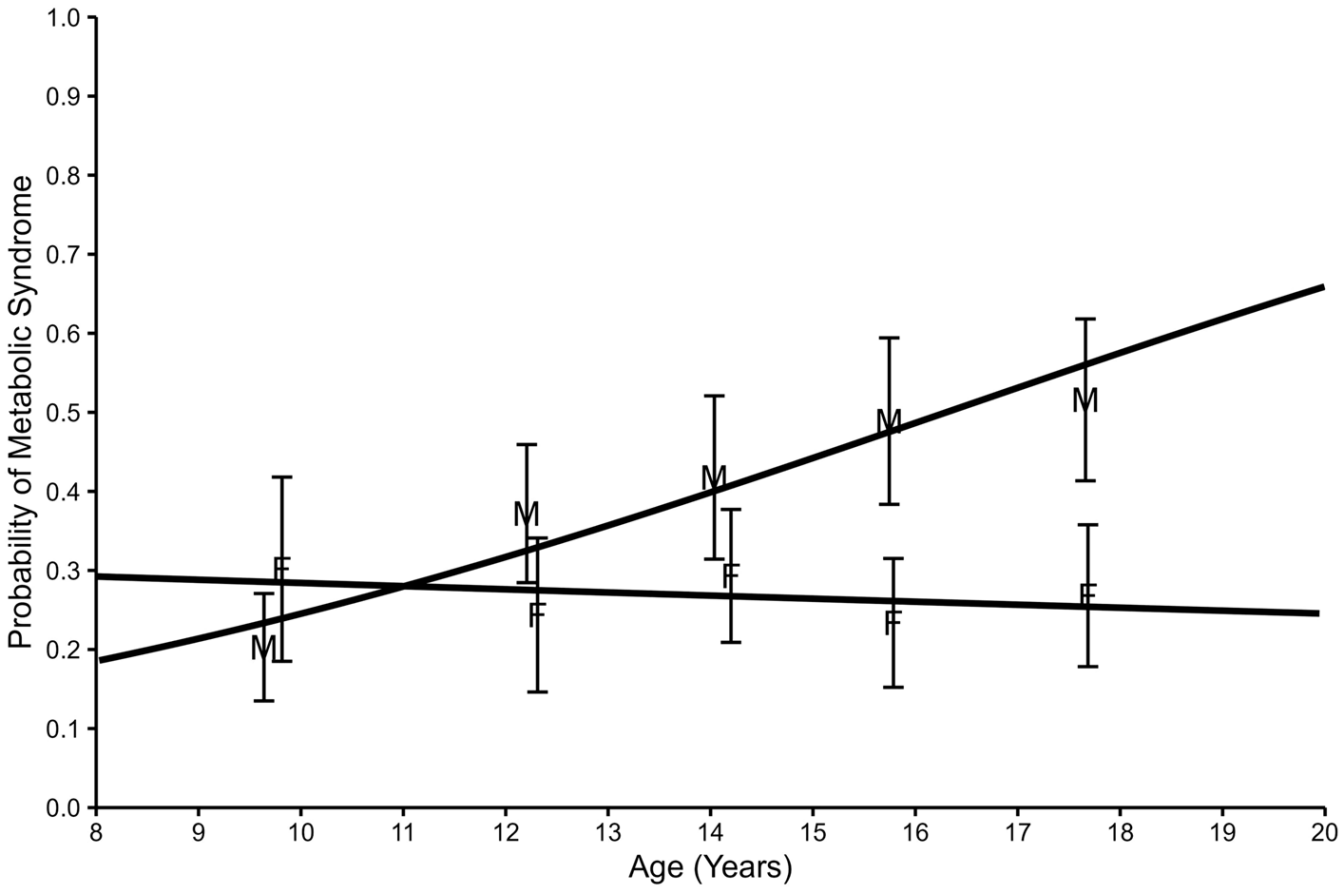
Predicted probability of metabolic syndrome by age and sex

Table 5a–c shows associations of MetS with demographic factors. Both age and sex were strongly associated with prevalence of MetS. Male vs female odds ratio (OR) was 1.7 (p < 0.001) for MetS. The odds of MetS increased rapidly with age in boys, but no association with age was evident among girls. In boys aged 14–15 years, 49.5% met criteria for MetS vs 26.0% for girls, and this difference increased with age with 66.7% boys aged 18–19 years with MetS vs 24% for girls. See Fig. 2 for the predicted probability of MetS by age and sex.

**Table 5:** (a) Metabolic syndrome and association with age, (b) Metabolic syndrome and association with sex, (c) Metabolic syndrome and association with race and ethnicity

(a)																		
	Metabolic syndrome			HDL < 40 mg/dL			Triglycerides ≥ 110 mg/dL			BMI ≥ 95th%ile for age-sex			Glucose ≥ 100 mg/dL			Sys BP or Dia BP ≥ 90th %ile for age-sex-height		
	Years	OR	p	Years	OR	p	Years	OR	p	Years	OR	p	Years	OR	p	Years	OR	p
Mean age Criterion not met	13.6			13.5			13.7			13.9			13.9			13.8		
Mean age Criterion met	14.3	1.09	< 0.001*	14.6	1.15	< 0.001*	14.1	1.06	0.013*	13.9	0.99	0.722	14.1	1.03	0.329	14.0	1.03	0.137

(b)																		
	Metabolic syndrome			HDL < 40 mg/dL			Triglycerides ≥ 110 mg/dL			BMI ≥ 95th%ile for age-sex			Glucose ≥ 100 mg/dL			Sys BP or Dia BP ≥ 90th %ile for age-sex-height		
	%	OR	p	%	OR	p	%	OR	p	%	OR	p	%	OR	p	%	OR	p
Female (F)– Ref cat	26.7			24.4			36.3			64.5			12.3			41.6		
Male (M)	38.1			35.8			45.6			66.7			16.1			48.2		
M vs F		1.7	< 0.001*		1.74	< 0.001*		1.48	0.003*		1.10	0.367		1.37	0.074		1.31	0.014*

(c)																		
	Metabolic syndrome			HDL-Cholesterol < 40mg/dL			Triglycerides ≥ 110 mg/dL			BMI ≥ 95th%ile for age-sex			Glucose ≥ 100 mg/dL			Sys BP or Dia BP ≥ 90th %ile for age-sex-height		
	%	OR	p	%	OR	p	%	OR	p	%	OR	p	%	OR	p	%	OR	p
White/ Caucasian –Ref cat	35.5			34.3		< 0.001*	47.0			64.4			14.5		0.981	45.4		
Black/AA	24.9			19.8			23.7			70.0			14.6			46.2		
White/Caucasian vs Black/AA		1.75	0.003*		2.13	< 0.001*			< 0.001*		0.775	0.080		0.990	0.981		0.971	0.833
Multiracial	29.4			22.6			36.2			62.9			13.9			42.1		
White/Caucasian vs Multiracial		1.33	0.293		1.79	0.025*		1.56	0.060		1.06	0.717		1.05	0.863		1.14	0.514
Other	35.2			33.3			38.9			70.3			11.5			44.0		
White/Caucasian vs Other		1.01	0.841		1.04	1.000		1.39	0.260		0.763	0.275		1.32	0.702		1.06	0.826
Non-Hispanic – Ref cat	32.6			30.6			41.2			65.1			14.1			46.0		
Hispanic	34.3			28.7			41.0			69.8			15.0			38.9		
Non-Hispanic vs Hispanic		0.926	0.726		1.10	0.694		1.01	0.961		0.806	0.190		0.926	0.792			0.081

*OR*, Odds ratio; *HDL*, high density lipoprotein-cholesterol; *BMI*, body mass index; *Sys BP*, systolic blood pressure; *Dia BP*, diastolic blood pressure, *Ref cat* Reference category, *AA* African American

*Statistical significance with p value < 0.05

White patients had significantly higher rates of MetS (35.5%) than Black/African American patients (24.9%) with OR = 1.75; p = 0.003. Patients identifying as Multiracial were intermediate between the White and Black/African American groups (although not being significantly different from either). There were no significant associations of MetS with Hispanic ethnicity.

MetS status was determinable for only 996 patients (63.6% of the sample) due to missing metabolic data. MetS criteria other than BMI were more likely to be collected from patients enrolled at inpatient sites. Patients with ≥ 1 lab based MetS data available vs those with none did not differ significantly regarding age, sex, or race (see Table 6). There were regional differences in laboratory data availability, with Northeast sites having much lower rates of available data. Hispanic ethnicity was statistically associated with no laboratory data (p = 0.003); however, this was attributable to regional differences as Hispanic patients were enrolled almost exclusively at Northeast sites. BMI (as a percentage of the 95th percentile for age and sex) was not significantly associated with odds of having laboratory data available.

**Table 6:** Demographic differences in patients with and without laboratory parameters

	≥ 1 Lab-Based MetS Criteria Measured (N = 1104)		No Lab-Based MetS Criteria Measured (N = 461)		
	Mean	SD	Mean	SD	p
Age (years), at randomization	13.9	2.9	13.9	2.8	0.745
	N	%	N	%	
Sex (assigned at birth)					0.470
Male	578	52.4	251	54.4	
Race					0.264
White/Caucasian	734	66.5	289	62.7	
Black/African American	203	18.4	87	18.9	
Multiracial	105	9.5	46	10.0	
Asian	22	2.0	9	2.0	
Native American	3	0.3	3	0.7	
Native Hawaiian/Pacific Islander	2	0.2	0	0.0	
Unknown	3	0.3	2	0.4	
Does not identify	32	2.9	25	5.4	
Ethnicity					
Hispanic origin	122	11.1	80	17.4	0.003*
Non-Hispanic	981	88.9	380	82.4	
Unknown	1	0.1	1	0.2	
	Mean	SD	Mean	SD	
Body Mass Measures					
BMI (kg/m^2^)	29.2	6.1	29.2	6.1	0.927
BMI z-score	1.86	0.48	1.86	0.46	0.991
BMI (as % of 95th percentile)	111.3	19.8	111.2	20.4	0.928
	**N**	**%**	**N**	**%**	
Obese	722	65.4	306	66.4	0.726

*SD* standard deviation, *BMI* body mass index

In the subgroup where all five criteria were measured, for those who were already OB, 46.2% had MetS, and for those who were OW, 6.4% had MetS (see Supplemental Table 1). The number of participants who had one additional criterion of MetS besides BMI was comparable in those who were OB at 39.7% versus those who were OW 35.3%. Similarly, 29.5% of participants with OB versus 20.7% of participants with OW met two additional MetS criteria besides BMI. This difference widens between OB and OW participants when three additional MetS criteria besides BMI are met with 15% in former and only 4.4% in latter group (see Supplemental Table 1).

### Components of metabolic syndrome

Boys were more likely to meet criterion for low HDL-cholesterol (OR = 1.74; p < 0.001), high triglycerides (OR = 1.48; p = 0.003), and elevated BP (OR = 1.31; p = 0.014). The HDL-cholesterol and triglycerides criteria were significantly associated with age and sex (p < 0.001 for HDL-cholesterol and p = 0.016 for triglycerides) in the same pattern as for MetS, indicating that these variables are the main contributors to those associations with MetS. The prevalence of dyslipidemia was different among racial and ethnic groups occurring in 47% White patients (n = 315/670), 27% Black/African American patients (n = 44/186), and 36% Multiracial patients (n = 34/94) with significant differences among these groups (White vs Black/African American p < 0.0001; White vs Multiracial p = 0.0598, Black/African American vs Multiracial p = 0.0341). There were no significant main or interaction effects regarding the BP or OB criteria. It should be noted that since the sample was selected based on relative body mass, the OB effect is only observed relative to those who are OW.

## Discussion

In this large pragmatic trial, the prevalence of MetS at baseline was about 33%. In the OB subpopulation, the prevalence of MetS was 41%, which is considerably higher than the prevalence of MetS of 29% in the general population of OB youth [17, 41]. The higher frequency of MetS in our OB subgroup was likely due to the additional effects of severe mental illness, genetics, unhealthy lifestyle behaviors, and antipsychotic treatment which each have additional adverse effects on cardiometabolic health [42–44]. The prevalence of MetS in the OW sample was lower in this study at 4.2% than the 11.9% reported in the systemic review by Friend and colleagues [17]. Our study’s definition of MetS, which utilized BMI ≥ 95th %ile instead of WC ≥ 90th %ile for age-sex, may undercount overweight participants with central adiposity. In the subgroup where all five criteria were measured, for those who were already OB, 46.2% had MetS, whereas for those who were OW, 6.4% had MetS. OB is a strong determinant of MetS status. Youth with OB and BSD likely have greater metabolic sequelae compared with youth with OB without mental illness due to the compounding of risk factors [42–45].

The prevalence of MetS in this study was higher than that reported in other samples of youth with bipolar disorders, with Mohite and colleague’s [18] study 14% and Li and colleague’s study 19.8% [6]. Our study had an enriched sample of already OW and OB youth so higher prevalence rates of MetS were to be expected. Almost half of the participants had been prescribed an SGA for over a year allowing more time for metabolic side effects to present. The prevalence of MetS in adults with bipolar disorder has been reported to be higher, 37.3% in patients not restricted to OW/OB in a large systematic literature review and meta-analysis by Vancampfort et al. [28].

Not surprisingly, OB was the most common criterion for predicting MetS, a finding that has been reported in other studies of youth with mood disorders [18, 41]. After body weight, elevated BP was the most common criterion met for MetS (45.1%) followed by elevated triglycerides (41.2%), and low HDL-cholesterol (30.5%). The frequency of BP elevations in this study (45.1%) was higher than in other studies examining MetS in youth [12, 35] likely because the BP criterion used to define MetS in this study was broader than in other definitions of metabolic syndrome. The proportion of patients in our study with elevated glucose criterion (14.3%) was like that reported by Li and colleagues for youth with bipolar disorder (15.4%). [6]

Our study demonstrated older white males to be most at risk for MetS. Previous studies have shown mixed sex differences. In a case–control study of adolescent inpatients with mood disorder diagnoses by Patel and colleagues, female patients had greater prevalence of MetS compared to males [46], and in a scoping review of MetS in the general population of children ages 6 to 12, prepubertal females had greater prevalence of MetS [47]. Whereas, the systematic review by Friend et al [17] and by Obita et al., [48] which included children and adolescents ages 2 to 18 years, males demonstrated greater prevalence of MetS. The odds of MetS increasing rapidly with age in males and not females may be related to increased comorbid disruptive behavior disorders in male participants. Disruptive behavior disorders, particularly attention-deficit/hyperactivity disorder (ADHD), are associated with greater odds of MetS [49]. We did not examine diagnostic correlates of MetS status, an analysis beyond the scope of this baseline study.

We found racial differences in prevalence rates of MetS. White patients had a significantly higher frequency of MetS than Black/African American patients, a finding that has been reported in other studies of MetS in youth with bipolar disorder [18, 46]. There were no significant associations of MetS with Hispanic ethnicity, likely the result of small sample size and limited data. Frequencies of dyslipidemia also varied by race, with White youth showing significantly higher frequencies of dyslipidemia than Multiracial or Black/African American participants. These findings align with previous studies showing a lower frequency of dyslipidemia in Black youth [19].

Metabolic parameters to identify MetS were available for 63.6% of our sample – a higher metabolic monitoring frequency compared to what has been published previously (less than 50% at baseline in routine outpatient care) [49]. Here, clinicians were prompted by study protocol to obtain laboratory tests after inclusion of the youth into the study. Of note, despite the instruction, one out of three overweight/obese youth with a BSD diagnosis and antipsychotic treatment, i.e., three concurrent risk factors, did not receive blood testing within the protocol’s flexible allowable time window.

Results of this study must be interpreted within its limitations. There is no consensus definition of MetS in youth, and our definition is not the same used in other studies. Given the pragmatic nature of the study, we utilized BMI%ile instead of WC%ile, which may have undercounted overweight youth with central adiposity. Hormonal changes during puberty can impact metabolic status, and puberty is associated with a marked decrease in insulin sensitivity [50]. We did not stratify participants based on age or pubertal status so this may be a significant confounder. We also did not stratify based on duration of SGA exposure and did not directly analyze the effect by time. These analyses were outside the purview of this report. We did not adjust for multiple comparisons to maximize statistical power in this secondary analysis.

Only 63.6% of the study sample had MetS data. Participants with at least one or more lab-based MetS criteria available versus those without any data were generally comparable. Hispanic ethnicity was associated with greater odds of having no laboratory data, but this finding should be interpreted with caution, as it is felt to represent a geographic difference. Laboratory testing was conducted under usual care conditions, so blood work may not have been obtained in the fasting state so there may have been upward bias with criterion inflating prevalence of MetS. Height, weight, and BP were obtained at each site by different study personnel. However, clear instructions were given regarding how to measure height and weight, and identical study stadiometers and weight scales were provided to each study site. Finally, a notable limitation was not being able to determine whether the barrier in obtaining laboratory work was the clinician failing to order necessary bloodwork, the patient/family not obtaining it, or the results not being provided to the clinician. Social determinants of health may play a role in attainment of metabolic monitoring and is a topic for further study. There are disparities in the prevalence of childhood obesity-related comorbidities with MetS risk increasing with low early-life socioeconomic status and with adverse childhood experiences [51, 52].

## Conclusion

In this large pragmatic trial of OW/OB youth with BSD treated with or newly initiated on an SGA, a third of the sample had MetS at baseline, increasing to 41% in OB youth and being most prevalent in boys and in White youth. The prevalence of MetS in the OB subgroup in this enriched sample was about one-third higher than the prevalence in nonclinical samples of OB youth. The overall MetS frequency was about 50–100% higher than what has been reported in previous studies of youth with mood disorders not enriched for OW or OB and treatment with SGAs. Just over 60% of the sample had necessary laboratory data to determine metabolic status, which underscores the importance of prescribers adhering to the metabolic monitoring guidelines in patients treated with SGAs. Prompt identification of MetS would allow for earlier interventions to reduce cardiometabolic burden either via healthy lifestyle interventions or pharmacological strategies [53–56] aiming to enhance patient outcomes. Future research is needed on effective interventions to mitigate metabolic abnormalities in these vulnerable youth.

## Supplementary Information

Below is the link to the electronic supplementary material.

Supplementary file1 (DOCX 20 kb)

## Data Availability

Data will be made available upon publication to researchers who provide a methodologically sound proposal for use in achieving the goals of the approved proposal. Proposals should be submitted to Dr. Klein at kleinci@UCMAIL.UC.EDU.

## Abbreviations

MOBILITY: Metformin for overweight and obese children and adolescents with bipolar spectrum disorders treated with second generation antipsychotics
MetS: Metabolic syndrome
BSD: Bipolar spectrum disorders
BMI: Body mass index
OW: Overweight
OB: Obese
WC: Waist circumference
