# Supplementary material for: Metabolic syndrome in youth with bipolar spectrum disorders treated with second-generation antipsychotics: baseline results from the community-based pragmatic MOBILITY Trial
Source: Eur Child Adolesc Psychiatry. 2025 Apr 11;34(9):2917–29. doi: 10.1007/s00787-025-02680-2 (PMC12507931; doi:10.1007/s00787-025-02680-2)
Supplement: Supplementary file 1 — Supplementary file1 (DOCX 20 kb) [file 787_2025_2680_MOESM1_ESM.docx]

**Supplemental Table 1. Number of criteria met based on weight status^1^**

| **Number of Criteria Met** | **N** | **%** | **Number of Additional Criteria Met**  **(if Obese)** | **N** | **%** |
| --- | --- | --- | --- | --- | --- |
| 5 | 10 | 1.1 |  |  |  |
| 4 | 94 | 10.7 | 4 | 10 | 1.7 |
| 3 | 186 | 21.1 | 3 | 88 | 15.0 |
| 2 | 268 | 30.4 | 2 | 173 | 29.5 |
| 1 | 226 | 25.6 | 1 | 207 | 35.3 |
| 0 | 98 | 11.1 | 0 | 109 | 18.6 |
|  |  |  |  |  |  |
|  |  |  | **Number of Additional Criteria Met**  **(if NOT Obese)** | **N** | **%** |
|  |  |  |  |  |  |
|  |  |  | 4 | 6 | 2.0 |
|  |  |  | 3 | 13 | 4.4 |
|  |  |  | 2 | 61 | 20.7 |
|  |  |  | 1 | 117 | 39.7 |
|  |  |  | 0 | 98 | 33.2 |

| ^1^Numbers are computed only from the subgroup with all 5 criteria measured |
| --- |
